# Vaccinology in pediatric rheumatology: Past, present and future

**DOI:** 10.3389/fped.2022.1098332

**Published:** 2023-01-10

**Authors:** Masa Bizjak, Merav Heshin-Bekenstein, Marc H. A. Jansen, Amit Ziv, Saskya Angevare, Yosef Uziel, Nicolaas M. Wulffraat, Natasa Toplak, for the PReS Vaccination Working Party

**Affiliations:** ^1^ Department of Allergology, Rheumatology and Clinical Immunology, University Children’s Hospital, University Medical Centre Ljubljana, Ljubljana, Slovenia; ^2^ Pediatric Rheumatology Service, Dana-Dwek Children’s Hospital, Tel Aviv Sourasky Medical Center, Tel Aviv, Israel; ^3^ Sackler Faculty of Medicine, Tel Aviv University, Tel Aviv, Israel; ^4^ Department of Pediatric Immunology and Rheumatology, Wilhelmina Children’s Hospital, University Medical Centre Utrecht, Utrecht, Netherlands; ^5^ Pediatric Rheumatology Unit, Department of Pediatrics, Meir Medical Center, Kfar Saba, Israel; ^6^ European Network for Children with Arthritis, Geneva, Switzerland; ^7^ KAISZ, Amsterdam, Netherlands; ^8^ Faculty of Medicine, Utrecht University, Utrecht, Netherlands; ^9^ Faculty of Medicine, University of Ljubljana, Ljubljana, Slovenia

**Keywords:** vaccination, pediatric rheumatology, safety, immunogenicity, biologic therapy

## Abstract

With the introduction of biological disease-modifying antirheumatic drugs (bDMARDs), the treatment of pediatric patients with autoimmune/inflammatory rheumatic diseases (pedAIIRD) has advanced from the “Stone Age” to modern times, resulting in much better clinical outcomes. However, everything comes with a price, and use of new bDMARDs has resulted in an increased risk of infections. Therefore, preventing infections in pedAIIRD patients is one of the top priorities. The most effective preventive measure against infection is vaccination. The first study on humoral immunity after vaccination in pediatric rheumatology was published in 1974 and on safety in 1993. For many years, data about safety and immunogenicity in pedAIIRD patients were available only for non-live vaccines and the first studies on live-attenuated vaccines in pedAIIRD patients treated with immunosuppressive therapy were available only after 2007. Even today the data are limited, especially for children treated with bDMARDs. Vaccinations with non-live vaccines are nowadays recommended, although their long-term immunogenicity and efficacy in pedAIIRD patients are still under investigation. Vaccinations with live-attenuated vaccines are not universally recommended in immunosuppressed patients. However, measles-mumps-rubella booster and varicella zoster virus vaccination can be considered under specific conditions. Additional research is needed to provide more evidence on safety and immunogenicity, especially regarding live-attenuated vaccines in immunosuppressed patients with pedAIIRD. Due to the limited number of these patients, well-designed, prospective, international studies are needed. Further challenges were presented by the COVID-19 pandemic. This mini review article reviews past and present data and discusses the future of vaccinology in pediatric rheumatology.

## Introduction

New treatments, including biological disease-modifying antirheumatic drugs (bDMARDs), have significantly increased survival and improved outcomes in pediatric patients with autoimmune/inflammatory rheumatic diseases (pedAIIRD). With advances in treatment, infections remain an important cause of morbidity and mortality in these patients, who are at increased risk of infection due to immune-modulating therapy as well as the underlying disease process (1–5). Many of these infections are vaccine preventable, so vaccination is even more important in patients with pedAIIRD compared to the healthy population, to achieve the highest possible protection. The first study on humoral immunity after vaccination in pediatric rheumatology was published in 1974 and on safety in 1993 (6, 7). For many years, data on safety and immunogenicity were available only for non-live vaccines. The first studies on safety and immunogenicity of live-attenuated vaccines in patients with pedAIIRD treated with immunosuppressive therapy were available only after 2007 (8). Since then, the evidence for vaccine safety and immunogenicity in patients with pedAIIRD has grown substantially and was recently summarized in the updated European League Against Rheumatism (EULAR)/Paediatric Rheumatology European Society (PReS) recommendations for vaccination of patients with pedAIIRD (9).

Despite the increasing evidence for vaccine safety and immunogenicity in patients with rheumatic diseases, vaccination coverage in pedAIIRD patients remains suboptimal, especially among patients treated with bDMARDs (10–12). A Canadian study on 200 children with juvenile idiopathic arthritis (JIA) reported complete vaccination coverage in 52%, 68% and 61% of patients at 2.5 years, 10.5 years and their last clinic visit, respectively, compared to 85% in 2-year-old healthy children (12). In a Slovenian study on 187 patients with pedAIIRD, vaccination coverage was comparable to the general population at 5 years but lower than in general population thereafter, with only 64.7% of patients having a complete vaccination status at their last clinic visit. Coverage rates for the two most commonly omitted vaccines, the 2nd dose of measles-mumps-rubella (MMR) vaccine and the hepatitis B virus (HBV) vaccine, were 61% and 59% in children treated with bDMARDs compared to 94% and 87% in general population, respectively (10). In a German study on 715 children with JIA, every third patient was incompletely vaccinated. Vaccination coverage was comparable to the general population in preschool children and lower in older children, for example 24%/79% for tetanus and diphtheria compared to 46%/95% of the general population. However, vaccination coverage for MMR at 60%–75% was comparable to the general population (11). Some rheumatic diseases can present in the first years of life and the complexity of the disease, together with current treatment strategies that include early use of immunosuppressive and biologic therapy, and fear of inducing relapse or a vaccine strain infection after vaccination with live-attenuated vaccines can add to uncertainty and lead to substantial differences in vaccination practices in patients with pedAIIRD (11, 13).

In this mini-review, we summarize the current knowledge and latest recommendations for live-attenuated and non-live vaccines in patients with pedAIIRD, including vaccination against coronavirus disease 2019 (COVID-19). Studies included in the first and the updated EULAR recommendations for vaccination of patients with pedAIIRD were considered together with more recent studies on this topic and on COVID-19 vaccination in patients with pedAIIRD (9, 13, 14). Furthermore, we discuss the challenges and future of vaccinology in pediatric rheumatology.

## Different vaccination practices across the globe

National Immunization Programs (NIP), parental obligation to vaccinate their children and vaccination coverage rates vary greatly among countries, which poses a challenge for global initiatives for uniform vaccination practices in children with pedAIIRD treated with immunosuppressive and immunomodulatory therapy (15, 16). About half of the European countries have mandatory childhood vaccinations, including Croatia, Hungary, Latvia, Poland, Serbia, Slovakia, Slovenia and Ukraine (17). France and Italy are among the countries that have recently strengthened and expanded their mandatory vaccination laws due to decreasing vaccination coverage and have seen increasing vaccination rates after changes in the law (18). The Czech Republic, Germany and Greece, as well as the United States of America, have mandatory vaccinations for school entry. Most countries in Latin America, including Brazil, have mandatory vaccinations, while they are recommended in Australia, Canada, India and Israel (17). Furthermore, vaccine policies can vary at the state level. For example, in some parts of Australia and Canada childhood vaccinations are required to access early childhood services and enter school, and in Australia to obtain family assistance benefits (19, 20). Varicella vaccination is not included in the mandatory vaccination programs of most countries (21).

Vaccination schedules and coverage differ among countries. The first dose of MMR vaccine is recommended at 11–15 months of age and most countries recommend the second dose before 2 years of age or before school entry at 5–6 years. However, the vaccination is administered at 3–4 years in a few countries and in some, including Hungary, the Netherlands, Estonia, Norway and Poland at 9 years or even later (22, 23).

Mandatory programs do not always ensure higher vaccination coverage. For example, the childhood vaccination rate is high (around 95%) in Australia, Israel, Norway, and Portugal, where vaccinations are recommended (24, 25). However, in many countries with mandatory vaccination, the coverage rate for MMR fell below the 95% that is necessary to achieve herd immunity, including Brazil, Croatia, The Czech Republic, Serbia and Slovenia (16, 25–27). This is in part probably due to increasing vaccination hesitancy in patients with chronic disorders, such as JIA. Vaccination coverage was further affected by the COVID-19 pandemic (28–30).

## Vaccinations with non-live vaccines in pediatric rheumatology

Administration of non-live vaccines to children with rheumatic diseases is recommended, regardless of the therapy they are receiving (9, 13). Many studies have demonstrated that non-live vaccines are safe in patients with pedAIIRD; however, long-term immunogenicity has been questioned. It is likely that both the disturbed immune system in patients with AIIRD and the immunosuppressive therapy they receive affect the production of protective antibodies. In patients with juvenile-onset systemic lupus erythematosus (jSLE) the response to vaccination was reduced when disease activity was high at the time of vaccination (31). Patients treated with rituximab (RTX), an anti-CD20 agent, exhibit reduced humoral response to vaccines; however, a T cell response can be induced (32). The trend towards lower immunity with time, lower seroprotection rates and lower levels of specific antibodies after vaccination in children treated with tumor necrosis factor inhibitor (TNFi) has been reported by a few studies (33–35). Other studies found no effect of TNFi therapy on immunogenicity (36–39).

A recently published systematic literature review on efficacy, immunogenicity and safety of vaccinations in patients with pedAIIRD that was the basis for the updated EULAR/PReS recommendations found 41 studies that investigated non-live vaccines in the last 11 years, from 2011 to 2021 (14). Few studies that primarily evaluated immunogenicity and safety also addressed efficacy. The largest number of studies (*n* = 14) concerned influenza vaccine; three of which also described infection rates. There was no significant difference between vaccinated patients with pedAIIRD and healthy controls in the number of children with influenza infection and importantly, vaccinated children with systemic-onset JIA experienced influenza-like illness less often compared to unvaccinated children (34, 40, 41). Five studies were published on the immunogenicity and safety of HBV and four on hepatitis A virus vaccine. Studies included mainly patients with JIA and jSLE and none assessed efficacy. The concentration of antibodies was lower in patients with JIA and jSLE, and seroprotection rates were either comparable to healthy controls or lower in a few studies (14). It was recently published that mycophenolate mofetil may have a negative effect on seroconversion after a primary HBV vaccination in children with rheumatic diseases. An additional booster dose in those children induced seroprotection (42). Five studies were published for pneumococcal infections, four for human papilloma virus (HPV) vaccination, three for tetanus, two for diphtheria, one for pertussis and one for meningococcal infection (14). There was a report of pneumococcal infection in one patient on RTX and one on TNFi, regardless of vaccination status (43, 44).

Except for pneumococcal vaccination in patients with cryopyrin-associated periodic syndrome (CAPS), vaccinations were safe: no serious adverse events were reported. Studies that reported disease activity found no increase after vaccination. Among CAPS patients, mainly in adults, cases of severe systemic reactions were described after 23-valent pneumococcal polysaccharide vaccine (45, 46).

## Vaccinations with live-attenuated vaccines in pediatric rheumatology

Administration of live-attenuated vaccines to patients with pedAIIRD has been a matter of debate. Main concerns are the risk of disseminated infection with a vaccine-strain pathogen, potential flare of underlying rheumatologic condition, and inadequate immunologic response to the vaccination due to immunosuppressive treatment.

The first study on safety and immunogenicity of live-attenuated vaccines in patients with pedAIIRD treated with immunosuppressive therapy was published in 2007 and the first prospective study in 2009 (8, 47). Since then, data on the safety and immunogenicity of live attenuated vaccines in this population have accumulated. In addition, the use of bDMARDs has increased significantly in the last two decades; therefore, data on the safety and immunogenicity in relation to these drugs have become available.

A randomized, open-label controlled trial observed good immunogenicity after the booster dose of MMR vaccine in JIA patients [including those taking methotrexate (MTX) and bDMARDs], no disseminated infection with the wild-type virus and no increase in disease flares in the year following vaccination (48). A multicenter retrospective study including 234 patients demonstrated that measles-mumps-rubella-varicella (MMR/V) booster vaccines were safe for pedAIIRD patients on immunosuppressive therapy. This is currently the largest dataset on the safety of the MMR/V booster in this population (49). Other studies have reported that MMR booster vaccine in pedAIIRD patients treated with immunosuppressive drugs was safe and immunogenic, although the antibody response was lower in the short- and longer-term (8, 50, 51). There are almost no data on primary MMR vaccination in immunosuppressed children as the first dose of this vaccine is typically given before the onset of most autoimmune disorders.

Several retrospective and prospective studies evaluated the safety and immunogenicity of primary and booster VZV vaccines in pedAIIRD patients treated with immunosuppressive and immune-modulating therapy. There were no complicated or disseminated varicella infections. Few patients developed a vaccine-induced mild and transient VZV-like rash, of which one was admitted for intravenous acyclovir treatment for safety reasons. While immunogenicity results were good, the vaccine was not efficacious in all children, as six patients who presented with breakthrough varicella infection were reported (52–57).

A few studies evaluated the safety and immunogenicity of the yellow fever (YF) vaccine in adult patients receiving immune-modulating treatment (58–60). The vaccine was found to be safe and immunogenic with a trend towards a lower immune response in patients treated with immunomodulation (59, 60). The first study evaluating safety of the YF vaccine in pedAIIRD patients on low dose immunosuppressive therapy that was published in December 2021 reported fractional dose of 17DD YF vaccine to be safe and immunogenic (61). Thus far, no studies have been conducted regarding the BCG vaccine and live typhoid vaccine in pedAIIRD patients. Data on live polio vaccination in these children are limited. One case described a 2-year-old boy with systemic-onset JIA treated with tocilizumab who had mild diarrhea after the vaccine (55). Rota virus vaccine is usually not relevant, as it should not be given after the age of 6 months.

A comprehensive review on vaccination in children on bDMARDs reported 136 patients treated with biologics while receiving a live-attenuated vaccine. This review showed that a booster of live-attenuated vaccines in children treated with bDMARDs was safe, but not always immunogenic, especially over the long term (15).

## COVID-19 and vaccination, what is already known

Even though hospitalizations for COVID-19 among children and young people with AIIRD were found to be rare and treatment with bDMARDs was not associated with more severe COVID-19, patients with pedAIIRD could be at risk for disease flare secondary to severe acute respiratory syndrome coronavirus 2 (SARS-CoV-2) infection or to withholding anti-inflammatory therapy during the COVID-19 illness (62, 63). While vaccination can protect against COVID-19, safety and immunogenicity data regarding COVID-19 vaccines among children with AIIRD are limited. An international, multicenter study, evaluating the safety and immunogenicity of the BNT162b2 anti-SARS-CoV-2 vaccine among 91 adolescents and young adults with juvenile-onset AIIRD, reported a good safety profile, with 96.7% of patients reporting mild or no adverse events, no change in disease activity scores, and 3.3% with transient acute symptoms. The seropositivity rate was 97.3% in the AIIRD group compared to 100% among healthy controls. However, anti-S1/S2 antibody titers were significantly lower in the AIIRD group than in controls (242 ± 136.4 vs. 387.8 ± 57.3 BAU/ml, respectively; *p* < 0.0001). No cases of COVID-19 were documented during the 3-month follow-up (63). A study on 159 adolescents and young adults with AIIRD concluded that the SARS-CoV-2 mRNA vaccines were efficacious after the two-dose regimen in almost all patients, without serious adverse events. The rate of disease flare was 4.4% after the second mRNA vaccine dose (64). A study that examined the BNT162b2 mRNA vaccine in 41 adolescents with AIIRD reported that patients who received immunomodulatory treatments safely achieved an effective humoral response after two-dose regimen vaccine, without interrupting their current treatments (65). A 2022 publication reported a good safety profile in a cohort of 228 children aged 12–18 years (66). Another study that evaluated the effectiveness of the BNT162b2 mRNA COVID-19 vaccine in preventing COVID-19 infection in a large cohort of 1,639 adolescents with pedAIIRD compared to healthy adolescents, showed that the vaccine was effective against COVID-19 infection, similar to healthy controls (67). These results should encourage vaccination of adolescents with juvenile-onset AIIRD, also while on immunomodulation.

## Current recommendations for vaccination of patients with pedAIIRD

Update of the 2011 EULAR vaccination recommendations for patients with pedAIIRD was published in June 2022 (9, 13). Since the first recommendations from 2011, several new studies were performed on the safety, immunogenicity and efficacy of both live-attenuated and non-live vaccines.

Recommendations were developed by an expert committee using the EULAR standard operating procedures. After a systematic literature review, 6 overarching principles and 7 recommendations were formulated and provided with the level of evidence, strength of recommendation and Task Force level of agreement.

First of all, the treating specialist should assess patients’ vaccination status annually. In general, the NIP should be followed for all patients with pedAIIRD, including live-attenuated vaccines under specific conditions. If possible, vaccinations should be administered during quiescent disease and prior to immunosuppressive treatment, but necessary treatment should never be postponed. Mainly, seroprotection is preserved in immunosuppressed patients receiving vaccinations, except for those on high dose glucocorticoids and B-cell depleting therapies. Some vaccines within the NIP require specific attention: the (10- or 13-valent) pneumococcal conjugate vaccine is recommended for all pedAIIRD patients and vaccination against HPV is advocated for non-vaccinated jSLE patients. In addition, the MMR booster can be administered safely to patients on MTX and can even be considered in patients on glucocorticoids and specified bDMARDS (TNFi, anti-IL1 and anti-IL6 therapy) (9, 48). Finally, additional vaccinations not included in the NIP as standard may be considered: The yearly influenza vaccination should be strongly considered in all pedAIIRD patients and physicians should strongly consider the VZV vaccination in varicella naive patients, also whilst on MTX. They also should consider this vaccination in naive patients on low dose glucocorticosteroids, TNFi, anti-IL1 and anti-IL6 therapy. Due to insufficient evidence in pediatric population, YF vaccination should be avoided in all immunosuppressed pedAIIRD patients.

These recommendations provide guidance to practitioners in daily practice to attain optimal infection prevention in immunocompromised patients with pedAIIRD (9).

## What will the future bring?

Pediatric rheumatology is developing rapidly, from expanding into new areas, such as the relatively new field of autoinflammatory diseases in children, to increasing recognition of involvement of other organ systems apart from the musculoskeletal system and shifting to earlier, more aggressive treatment with increasing use of bDMARDs and the emergence of new drugs, including targeted synthetic DMARDs/JAK inhibitors. With these new approaches, infection prevention remains one of the crucial points in the treatment of pedAIIRD patients. The updated recommendations for vaccination of patients with pedAIIRD strengthen the role of the treating specialists in the field of vaccinology by empowering them to check and update their patients’ vaccination status. They also strengthen the recommendations for using live-attenuated MMR booster and VZV vaccines in immunosuppressed patients (9, 14). These new approaches may increase vaccine awareness for all pedAIIRD patients, contribute to more uniform vaccination practices, and hopefully increase vaccination coverage, which is currently suboptimal. Well-designed, prospective, multicenter, international studies are needed due to the limited number of patients with pedAIIRD. One such study on the safety and immunogenicity of MMR booster vaccine in patients on immunosuppressive treatment, including bDMARDs, is currently recruiting patients. On the other hand, new vaccines with more favorable safety profile will probably emerge in the future. One of such vaccines, the non-live VZV vaccine, is already available and can be especially important for immunocompromised patients, but studies are yet to come. New platforms, such as the development of mRNA based COVID-19 vaccines, are also an emerging opportunity.

There are still many questions to be answered, including long-term protection after vaccination in patients with pedAIIRD, the need for booster doses, the ideal time for vaccination, and the effect on immunogenicity and safety of the vaccine regarding the timing of vaccination and drug application.

## Conclusion

Evidence for the safety of vaccinations in patients with pedAIIRD is growing. However, immunogenicity is lower among patients treated with specific immunosuppressive drugs as compared to healthy population. Immunization practices vary greatly among countries and the current COVID-19 pandemic era presents some challenges for vaccinology in pediatric rheumatology. The updated recommendations for vaccination of pedAIIRD patients provide guidance to practitioners in daily practice, to attain optimal infection prevention and emphasize the importance of a tailor-made vaccination schedule for these patients, considering their disease activity, treatment, infection risk, vaccine safety and efficacy. Well-designed, prospective, multicenter, multinational studies are needed due to the limited number of patients with pedAIIRD to strengthen the recommendations for vaccinations in these patients.
